# Pluripotent Nontumorigenic Adipose Tissue‐Derived Muse Cells have Immunomodulatory Capacity Mediated by Transforming Growth Factor‐β1

**DOI:** 10.5966/sctm.2016-0014

**Published:** 2016-08-02

**Authors:** María L. Gimeno, Florencia Fuertes, Andres E. Barcala Tabarrozzi, Alejandra I. Attorressi, Rodolfo Cucchiani, Luis Corrales, Talita C. Oliveira, Mari C. Sogayar, Leticia Labriola, Ricardo A. Dewey, Marcelo J. Perone

**Affiliations:** ^1^ Instituto de Investigación en Biomedicina de Buenos Aires, National Scientific and Technical Research Council (CONICET), Partner Institute of the Max Planck Society, Buenos Aires, Argentina; ^2^ Servicio de Cirugía Plástica, Hospital Austral, Derqui, Argentina; ^3^ Biochemistry Department, Chemistry Institute, University of São Paulo, São Paulo, Brasil; ^4^ Cell and Molecular Therapy Center (Núcleo de Terapia Celular e Molecular/NETCEM), School of Medicine, University of São Paulo, São Paulo, Brasil; ^5^ Laboratorio de Terapia Génica y Células Madre, Instituto de Investigaciones Biotecnológicas–Instituto Tecnológico de Chascomús (IIB‐INTECH), National Scientific and Technical Research Council, National University of General San Martin, Chascomús, Argentina

**Keywords:** Spheroids/clusters, Stem cells, T lymphocytes, Antigen‐specific response, Immunomodulation

## Abstract

Adult mesenchymal stromal cell‐based interventions have shown promising results in a broad range of diseases. However, their use has faced limited effectiveness owing to the low survival rates and susceptibility to environmental stress on transplantation. We describe the cellular and molecular characteristics of multilineage‐differentiating stress‐enduring (Muse) cells derived from adipose tissue (AT), a subpopulation of pluripotent stem cells isolated from human lipoaspirates. Muse‐AT cells were efficiently obtained using a simple, fast, and affordable procedure, avoiding cell sorting and genetic manipulation methods. Muse‐AT cells isolated under severe cellular stress, expressed pluripotency stem cell markers and spontaneously differentiated into the three germ lineages. Muse‐AT cells grown as spheroids have a limited proliferation rate, a diameter of ∼15 µm, and ultrastructural organization similar to that of embryonic stem cells. Muse‐AT cells evidenced high stage‐specific embryonic antigen‐3 (SSEA‐3) expression (∼60% of cells) after 7–10 days growing in suspension and did not form teratomas when injected into immunodeficient mice. SSEA‐3^+^‐Muse‐AT cells expressed CD105, CD29, CD73, human leukocyte antigen (HLA) class I, CD44, and CD90 and low levels of HLA class II, CD45, and CD34. Using lipopolysaccharide‐stimulated macrophages and antigen‐challenged T‐cell assays, we have shown that Muse‐AT cells have anti‐inflammatory activities downregulating the secretion of proinflammatory cytokines, such as interferon‐γ and tumor necrosis factor‐α. Muse‐AT cells spontaneously gained transforming growth factor‐β1 expression that, in a phosphorylated SMAD2‐dependent manner, might prove pivotal in their observed immunoregulatory activity through decreased expression of T‐box transcription factor in T cells. Collectively, the present study has demonstrated the feasibility and efficiency of obtaining Muse‐AT cells that can potentially be harnessed as immunoregulators to treat immune‐related disorders. Stem Cells Translational Medicine *2017;6:161–173*

**Significance Statement**

The present study reports on a simple, low‐cost, and reproducible method for the generation of multilineage‐differentiating stress‐enduring cells from human adipose tissue lipoaspirates without the aid of cell sorting and genetic manipulation methods. This stem cell population does not form teratoma in vivo when injected into immunodeficient mice and has immunomodulatory properties with potential therapeutic effects on immune disorders.

## Introduction

Stem cells in their natural physiological niche contribute to the functional maintenance of organs, tissue remodeling and repair, and cell renewal 1, 2. In conjunction with a better understanding of the functional properties within a normal physiological context, knowledge regarding the ability to differentiate into various cell types resulted in the classification of different stem cell types. Thus, multipotent stem cells, including mesenchymal stromal cells (MSCs), differentiate into all cell types from a specific germ layer 3. In contrast, embryonic stem (ES) cells and induced pluripotent stem cells (iPSCs) possess the potential to give rise to cells of all three germ lineages (e.g., mesodermal, endodermal, and ectodermal) 4, 5. The use of cell therapy to repair damaged tissue has been fueled during recent years by the fervent advancement of stem cell research. However, concerns have arisen regarding the use of pluripotent ES cells for regenerative medicine. This has mainly been attributed to teratoma formation through their uncontrolled self‐renewal and triploblastic differentiation. Additionally, their use has been limited because of ethical controversy. Although the use of iPSCs resolved the bioethical issues concerning ES cells, teratoma formation on transplantation has impeded their use in regenerative medicine.

MSCs can be isolated from a variety of tissues and expanded in vitro. MSCs from bone marrow aspirates are good candidates for cell replacement therapy mainly because of their accessibility, abundance, and nontumorigenic activity. MSCs have been used in several clinical trials 6 7 8; however, their use has been limited by the low rates of implantation. Recently, a population of pluripotent stem cells has been isolated from human adult dermal fibroblasts and bone marrow stromal cells retaining self‐renewing capacity despite their lack of teratoma formation when injected into immune‐compromised mice. These cells have been termed multilineage‐differentiating stress‐enduring (Muse) cells. This cell population is stress tolerant and consistently expresses pluripotency markers such as Nanog, Oct3/4, and Sox2 9, 10. In addition, Muse cells will home to damaged tissue in vivo and spontaneously differentiate into tissue‐specific cells when infused into the blood stream 9. Recent studies have shown the efficacy of Muse cells in generating dermal and epidermal cells in skin ulcers of diabetic immunodeficient mice 11 and to generate neural cells in stroke animal models 12.

In 2013, the isolation of Muse cells from human adipose tissue (Muse‐AT) with similar features to their fibroblast‐ and bone marrow‐derived counterparts was reported 13. Obtaining subcutaneous adipose tissue from adult humans by lipoaspiration is a safe and noninvasive procedure 14. Heneidi et al. developed a novel method for the isolation of Muse‐AT cells, taking advantage of their stress endurance properties 13. Muse‐AT cells can differentiate into cells of the three germ lineages both spontaneously and by differentiation induction and demonstrate downregulation of genes involved in embryonic development. Hundreds of millions of Muse‐AT cells can be extracted from ∼1 to 2 liters of tissue. The isolation procedure is both time‐efficient and cost‐effective, without the need for cell sorting techniques, genetic manipulation, or long periods of cell culture. However, the results reported by Heneidi et al. 13 on the intrinsic pluripotent and nontumorigenic properties of Muse‐AT cells have yet to be validated by other investigators. Moreover, whether Muse‐AT cells possess an immunoregulatory capacity has not been examined. Suppression of an uncontrolled immune response by stem cell administration is a possible therapeutic strategy for immunological diseases. Previous studies have reported modulation of effector T and B lymphocytes, dendritic cells, macrophages and natural killer cells by MSCs and ES cells 15 17 18 19. In the present study, we used T lymphocytes that recognize a post‐translationally modified chromogranin A‐derived peptide, isolated from a transgenic CD4^+^T‐cell receptor nonobese diabetic (NOD) mouse strain (NOD BDC2.5) 20, 21. BDC2.5 T cells can be stimulated in vitro by mimotopes in the context of major histocompatibility complex class II complexes 22. The autoreactive BDC2.5 CD4^+^T clone resembles effector T cells of the T helper 1 (Th1) phenotype in that it produces interleukin‐2 (IL‐2), interferon‐γ (IFN‐γ), and tumor necrosis factor‐α (TNF‐α) on antigen stimulation. Thus, the BDC2.5 T clone is a very useful model for the study of antigen‐specific CD4^+^T lymphocyte regulation in Th1‐driven autoimmune diabetes 23.

In the present report, we describe the procurement of Muse cells derived from human adipose tissue by a simple procedure and demonstrate further enrichment of this population by culturing under nonadherent conditions. We have confirmed previous results showing that Muse‐AT cells isolated under severe cellular stress conditions (e.g., long‐term collagenase incubation, lack of nutrients, low temperature, and hypoxia) will group in spheroid structures, express pluripotency markers, differentiate into the three germline cells, and have a normal karyotype. In addition, Muse‐AT cells did not form teratomas after injection in immunodeficient mice. Furthermore, our studies offer new insight into the cellular structure and immunomodulatory characteristics of Muse‐AT cells, focusing on their ability to downmodulate T‐cell activation in vitro. We investigated the impairment of the inflammatory response using the cell line RAW 264.7 and freshly isolated murine peritoneal macrophages (MΦ) after lipopolysaccharide (LPS) challenge under the influence of Muse‐AT cells. Muse‐AT cells expressed pluripotent stem cell markers concomitantly with a spontaneous increase in TGF‐β1 expression. The TGF‐β1/phosphorylated SMAD2 (pSMAD2) signaling pathway seems to be the pivotal molecular mechanism responsible for the immunoregulatory capacity of Muse‐AT cells. Collectively, the present investigation offers a more in‐depth, diversified look into the promising potential use of Muse‐AT cells to treat immune diseases.

## Materials and Methods

### Mice and Reagents

NOD BDC2.5 transgenic T‐cell receptor (NOD BDC2.5) mice were originally purchased from Jackson Laboratories (Bar Harbor, ME, http://www.jax.org). C57BL/6J mice were obtained from our animal facility. All mice were bred in our animal facility and kept under semibarrier conditions. For all experiments, 8–10‐week‐old female mice were used in accordance with protocols approved by the Institutional Board on Animal Care, Facultad de Ciencias Exactas y Naturales (School of Exact and Natural Sciences), University of Buenos Aires. Collagenase type IA and SB 431542 (SB) were purchased from Sigma‐Aldrich (St. Louis, MO, http://www.sigmaaldrich.com); low‐glucose Dulbecco's modified Eagle's medium (DMEM) and Roswell Park Memorial Institute (RPMI) 1640 medium from Thermo Fisher Scientific Life Sciences (Waltham, MA, http://www.thermofisher.com), and fetal bovine serum (FBS) from Natocor (Cordoba, Argentina, www.natacor.com.ar).

### Isolation of Muse‐AT Cells From Human Abdominal Lipoaspirate Material

Human abdominal subcutaneous lipoaspirate material was processed in our laboratory 4–16 hours after elective plastic liposuction. The Institutional Evaluation Committee of Universidad Austral, Facultad de Ciencias Biomédicas, Derqui approved the procedure. The isolation procedure was performed as indicated, with some modifications 13. In brief, phosphate‐buffered saline (PBS)‐washed lipoaspirates (100–200 ml) were incubated with 0.1% (wt/vol) collagenase in DMEM for 30 minutes at 37°C with agitation, followed by further incubation at 4°C under hypoxic conditions for 16 hours, centrifugation at 340*g*, and discarding of the supernatants. The cell pellets were depleted of red blood cells by lysis with ammonium chloride and washed twice with PBS. The Muse‐AT cells (3.5 × 10^6^) were cultured in 60‐mm nonadherent plastic dishes in the presence of DMEM supplemented with 20% FBS and antibiotics and cultured in suspension for the indicated days. The growth media were changed every 3 days. Supernatants were collected by centrifugation (300*g* for 10 minutes at 4°C) and subsequently stored at −80°C until use.

### Culture of the Cell Line RAW 264.7 and Isolation of Peritoneal Macrophages

The mouse macrophage‐like cell line RAW 264.7 was cultured at 37°C in 5% CO_2_ in DMEM supplemented with 10% FBS growth medium containing 2 mM glutamine, 100 U/ml penicillin, and 100 mg/ml streptomycin (Thermo Fisher). Primary macrophages (Mϕ) were obtained from euthanized C57BL/6J mice after cervical dislocation. In brief, 10 ml of cold PBS was injected intraperitoneally into each mouse. After 5 minutes, peritoneal fluid was withdrawn slowly. The cells were centrifuged, washed twice, and cultured in RPMI 1640 plus 10% FBS at a cell density of 2 × 10^4^ per milliliter in 96‐well plates.

### Spontaneous Differentiation of Muse‐AT Cells Into Three Germline Cell Lineages

Spontaneous differentiation of Muse‐AT cells into mesodermal, endodermal, and ectodermal lineages was analyzed in adherent Muse‐AT cells after 7 days in culture by polymerase chain reaction (PCR) using primers for microtubule‐associated protein 2 (MAP‐2) as a marker of mesodermal cells, α‐fetoprotein for endodermal cell origin, and NK2 homeobox 5 (Nkx2.5) to identify neural‐like cells (ectodermal cell origin).

### Induced Differentiation of Muse‐AT Cells Into Three Germline Cell Lineages

Muse‐AT cells were seeded onto adherent dishes for induction into the three germline cell lineages. For myocyte induction, Muse‐AT cells were incubated with 5% *N*‐hydroxysuccinimide (NHS), 50 µM hydrocortisone, and antibiotics in DMEM supplemented with 20% FBS. Hepatocyte differentiation medium consisted of DMEM plus 20% FBS, 10 µg/ml insulin, 5 µg/ml transferrin, 7 ng/ml Na_2_SeO_3_, 10 nM dexamethasone, and 100 ng/ml hepatocyte growth factor‐4. For myocyte and hepatocyte induction, Muse‐AT cells were cultured in their respective differentiating media for 7 days, and their identity was revealed by immunofluorescence microscopy of smooth muscle actin (SMA) expression and cytokeratin‐7, respectively. For neuron formation, serum‐free neurobasal medium (Thermo Fisher) supplemented with B‐27, 2 mM glutamine, 30 ng/ml basic fibroblast growth factor (bFGF), and 30 ng/ml endothelial growth factor (both from Peprotech, Rocky Hill, NJ, http://www.peprotech.com) was used for 7 days. Next, the cells were cultured for an additional 7 days in DMEM plus 2% FBS, 25 ng/ml bFGF, and 25 ng/ml brain‐derived growth factor (Peprotech).

### Confocal Microscopy

Muse‐AT cells were harvested after the indicated time in culture, spun onto glass slides, and fixed by the addition of cold methanol. The cells were incubated with the following primary antibodies: anti‐stage‐specific embryonic antigen (SSEA‐3; BioLegend, San Diego, CA, http://www.biolegend.com), anti‐human embryonic cell marker panel (Oct‐4, Nanong, Sox‐2, Tra1‐60, and SSEA‐4; Abcam), and anti‐TGF‐β (Santa Cruz Biotechnology, Santa Cruz, CA, http://www.scbt.com). The secondary antibodies, used at 1 to 200 dilution, were either mouse or rabbit Alexa Fluor 647 conjugated dye (Thermo Fisher), rat Alexa Fluor 647 conjugated dye (Abcam), and mouse or rabbit Alexa Fluor 488 conjugated dye (Thermo Fisher). The slides were mounted with Mowiol (Sigma‐Aldrich). Images were acquired on an inverted Zeiss LSM 710 (Carl Zeiss Microscopy GmbH, Jena, Germany, http://www.zeiss.com). Data acquisition was performed using ZEN Black 2011 software (Carl Zeiss Microscopy) and quantification using Fiji (software. For identification of cell lineages, mouse anti‐human SMA (Thermo Fisher), rabbit anti‐human MAP‐2 (AbD Serotech [now Bio‐Rad Laboratories], Hercules, CA, http://www.bio-rad.com), and rabbit anti‐human α‐fetoprotein (Dako, Agilent Technologies, Santa Clara, CA, http:www.dako.com) were used, followed by secondary conjugated antibodies.

### Electron Transmission Microscopy

Clustered and single Muse‐AT cells were fixed in 2.5% glutaraldehyde containing PBS and postfixed in cold 1% osmium tetroxide at room temperature. The cells were then dehydrated in graded acetone, passed through propylene oxide, and embedded in Durcupan resin (Sigma‐Aldrich). Ultrathin sections were stained with uranyl 2% and Reynolds solution. Cell sections were examined using a Zeiss 109T electron microscope with a digital camera (Gatan ES 1000 W; Gatan, Pleasanton, CA, http://www.gatan.com).

### Antigen‐Specific T‐Cell Response In Vitro

Splenocytes from NOD BDC2.5 mice were obtained as previously described 23 and stimulated with the mimotope (Ac‐MVLPLWVRME‐NH_2_) during the time indicated in the presence or absence of conditioned media (CM) 24. Detection of IFN‐γ, TNF‐α, and IL‐10 in supernatants was performed according to manufacturer's protocol using enzyme‐linked immunosorbent assay sets (BioLegend). For T‐lymphocyte proliferation, splenocytes were stained with carboxyfluorescein‐diacetate succinimidyl ester (Fluka; Sigma‐Aldrich) cultured in RPMI, and stimulated with the mimotope for 72 hours. CD4^+^T lymphocytes were stained for fluorescence activated cell sorter (FACS) analysis with biotinylated anti‐CD4 (clone GK1.5), followed by streptavidin‐allophycocyanin (eBioscience, San Jose, CA, http://www.ebioscienc.com). Nonviable cells were excluded from analysis by 7‐aminoactinomycin D staining. The analysis was performed using FACS DIVA6 software (BD Biosciences).

### SDS‐Polyacrylamide Gel Electrophoresis and Western Blot Analysis

Splenocytes from NOD BDC2.5 mice were obtained and stimulated with the mimotope as described previously for 24 hours and then pretreated with vehicle and SB (10 μM) for 1 h, and Muse‐AT cell CM were added for 30 and 60 minutes. Cells were harvested on ice‐cold PBS, washed, and lysed in Laemmli sample buffer. Whole‐cell lysates were sonicated and heated to 95°C for 5 minutes. Proteins were subjected to SDS‐polyacrylamide gel electrophoresis and blotted with antibodies against pSMAD2 and SMAD2 (Cell Signaling Technologies, Danvers, MA, http://www.cellsignal.com), TBX21 (T‐box transcription factor; also known as T‐bet; Santa Cruz Biotechnology) and glyceraldehyde 6‐phosphate dehydrogenase (Abcam). Chemiluminescent signals were detected by horseradish peroxidase‐conjugated secondary antibodies and enhanced chemiluminescence (SuperSignal West Dura; Thermo Fisher) using a G:BOX Chemi XT4 (Syngene, Cambridge, UK, http://www.syngene.com). Quantification was performed digitally with ImageJ software (NIH, Bethesda, MD, http://www.imagej.nih.gov).

### Flow Cytometry Analysis of Muse‐AT Cells

Muse‐AT cells were incubated at 4°C for 30 minutes in the presence of the following fluorochrome‐conjugated antibodies: anti‐SSEA3‐Alexa Fluor 647 (BD Biosciences), anti‐CD105‐APC, anti‐CD90‐FITC, anti‐CD73‐PE, anti‐CD29‐PE, anti‐CD34‐PE, anti‐CD73‐PE, anti‐CD45‐FITC, anti‐HLA‐ABC‐PE, anti‐HLA‐DR‐PE, and anti‐CD44‐PE (all from BioLegend). As a negative control, isotype‐matched irrelevant monoclonal antibodies were used. Analysis (10^4^ events per run) was performed using the FACSCantoII flow cytometer and DIVA6 software (BD Biosciences).

### Determination of Karyotypes

Muse‐AT cells maintained in suspension cultures for 10–13 days were treated with quinacrine‐Hoechst staining, incubated in 0.075 M KCl, fixed in Carnoy's fixative, microphotographed, and analyzed (*n* = 3).

### Teratoma Assay in Immunodeficient Mice and Histological Analysis

Clusters of Muse‐AT cells were collected after 7–10 days in suspension cultures. The clusters were disrupted in single cells by trypsin, washed, suspended (10^6^ per 50 µl in PBS) and injected with a 30‐guage needle into the right testes of NOD*scid* mice. The P19 mouse embryonic carcinoma cell line was injected (10^6^) as a positive control into the left testes (*n* = 3). The mice were killed for analysis at 20, 60, 90, and 180 days after Muse‐AT cell injection. The testes were fixed in 4% paraformaldehyde, paraffin‐embedded, and stained with H&E.

### Qualitative and Quantitative Real‐Time PCR

Total RNA was extracted from AT‐Muse cells or splenocytes using TRI reagent (Sigma‐Aldrich). Complementary DNA (cDNA) synthesis was performed using Moloney murine leukemia virus reverse transcriptase in the presence of RNasin RNase inhibitor (Promega, Madison, WI, http://www.promega.com). The PCR primers were all intron spanning. cDNAs were amplified using Taq DNA polymerase (Thermo Fisher). Quantitative real‐time PCR was performed with SYBR Green I (Roche Life Science, Indianapolis, IN, http://www.lifescience.roche.com) using a CFX96 Touch Real‐Time PCR Detection System and the following sequences: human α‐fetoprotein, forward 5′‐CCACTTGTTGCCAACTCAGTGA‐3′, reverse 5′‐TGCAGGAGGGACATATGTTTCA‐3′; human MAP‐2, forward 5′‐ACTACCAGTTTCACACCCCCTTT‐3′, reverse 5′‐AAGGGTGCAGGAGACACAGATAC‐3′; human nkx2.5, forward 5′‐ CCCACGCCCTTCTCAGTCAA‐3′, reverse 5′‐GTAGGCCTCTGGCTTGAAGG‐3′; human hypoxanthine‐guanine phosphoribosyltransferase (HPRT), forward 5′‐CTCCGTTATGGCGACCCGCAG‐3′, reverse 5′‐GGCTACAATGTGATGGCCTCCCA‐3′; and human glyceraldehyde 6‐phosphate dehydrogenase, forward 5′‐TACTAGCGGTTTTACGGGCG‐3′, reverse 5′‐TCGAACAGGAGGAGCAGAGAGCGA‐3′. The mouse sequences used were as follows: GATA‐binding protein 3 (GATA‐3) forward 5′‐CTACCGGGTTCGGATGTAAGTC‐3′, reverse 5′‐GTTCACACACTCCCTGCCTTCT‐3; and IL‐10, forward 5′‐CTGGACAACATACTGCTAACCG, reverse ATTTCCGATAAGGCTTGGAAC. Relative expression was calculated for each gene using the Ct method with HPRT for normalization.

### Statistical Analysis

The results are presented as the mean ± SEM. Comparison between all the means was performed using analysis of variance followed by Bonferroni's multiple comparison test. A *p* value < .05 was considered to indicate a statistically significant difference. Analysis was performed using GraphPad Prism (GraphPad Software, Inc., La Jolla, CA, http://www.graphpad.com).

## Results

### Severe Stress Conditions Activate a Unique Human Adipose‐Derived Stem Cell Population Grown in Suspension Clusters In Vitro

**Figure 1:**
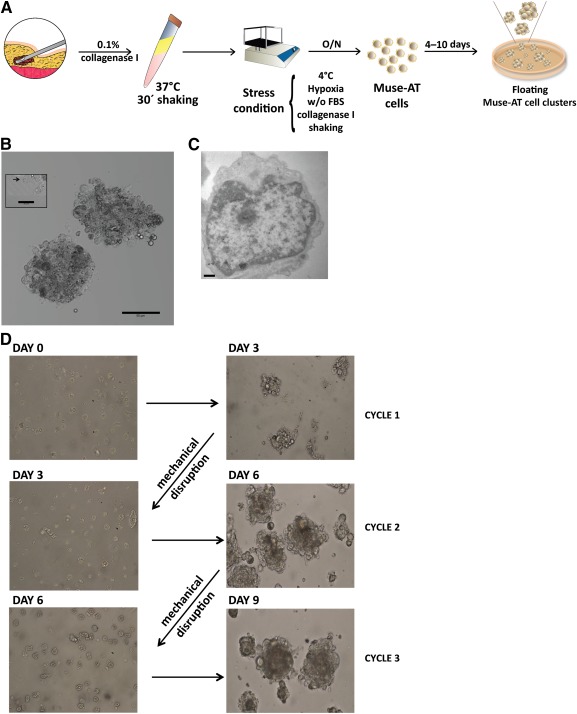
Generation and culture of liposuction‐derived Muse‐AT cells. **(A):** Scheme of Muse‐AT cell preparation protocol. Muse‐AT cells were obtained after digestion with collagenase and severe stress conditions. Clusters were generated when Muse‐AT cells were seeded on nonadherent plastic. **(B):** Muse‐AT cells formed clusters of 50–150 µm in diameter that grew in suspension culture. Scale bar = 50 µm. Left inset, black arrow indicates a single Muse‐AT cell at the edge of a cluster. Scale bar = 20 µm. **(C):** Transmission electron microscopy of a representative Muse‐AT cell after 5–7 days in culture showing a high nucleus to cytoplasm ratio. Scale bar = 500 nm. **(D):** Muse‐AT cells were seeded immediately on nonadhesive dishes after isolation (day 0) and formed clusters after 3 days of culture (day 3, cycle 1). After mechanical disaggregation, spheroids formed again, reaching 50–150 µm in diameter during the second and third growth cycle. Abbreviations: FBS, fetal bovine serum; Muse‐AT, multilineage‐differentiating stress‐enduring cells derived from adipose tissue; O/N, overnight; w/o, without.

Quiescent stem cells can be activated and released from their niche by the particular microenvironment generated when injured tissues respond to stress or damage 25. Under severe cellular stress conditions, Muse‐AT cells were obtained from adipose tissue aspirates following a previously described protocol 13, with some modifications. This procedure, which requires approximately 20 hours, allowed us to reach an average yield of 10^7^ cells (±0.25 × 10^7^; *n* = 10) per 100 g of lipoaspirate and avoiding the need for cell sorting techniques, genetic procedures, and long periods of culturing (Fig. 1A). Lipoaspirate material subjected to long incubation with collagenase without nutrients, low temperature, and hypoxic conditions released a homogeneous population of cells (10–15 µm in diameter) with a morphology resembling the previously characterized Muse‐AT cells 13. Muse‐AT cells displayed homogeneity in size and grew as floating undifferentiated single cells when freshly transferred into nonadherent plastic dishes.

Within 24 hours of culture, Muse‐AT cells spontaneously formed clusters of cells ( supplemental online Video 1) similar to that of ES‐derived embryoid bodies and bone‐marrow and dermal fibroblast‐derived Muse cells 9, 10. It has previously been suggested that the cluster size limited the cell division of Muse cells 9, 13. After 72 hours in culture, Muse‐AT cells formed tightly packed clusters that stopped growing when they reached an approximate diameter of 50–150 µm (Fig. 1B). After 5–7 days of culturing in nonadherent plastic, Muse‐AT cells showed an ultrastructural organization similar to that of ES cells, with large euchromatic nuclei and small cytoplasms as detected by transmission electron microscopy. In addition, Muse‐AT cells exhibited few cell surface extensions (Fig. 1C). To assess the ability of Muse‐AT cells to undergo self‐renewal, they were subjected to single‐cell suspension culture to obtain first‐round clusters. The clusters were disrupted mechanically, transferred to a new nonadherent culture dish, and allowed to form fresh clusters for 3 days. Second‐generation clusters were obtained by repeating the procedure one more time (Fig. 1D). Clusters were able to survive passaging onto new plates for up to four to five cycles at the most, at which point, they become resistant to mechanical dissociation into single cells (data not shown). These results confirmed that only Muse‐AT cells survived with severe cellular stress, and all other cells present in fat tissue (e.g., adipose‐derived stromal cells [ASCs], pre‐adipocytes, endothelial cells, macrophages, and fibroblasts) could not resist such harsh environmental conditions and died. Furthermore, Muse‐AT cells displayed the morphological and self‐renewal features of ES cells but with limited in vitro proliferation activity.

### Muse‐AT Cell Clusters Express Pluripotency Stem Cell Markers

**Figure 2:**
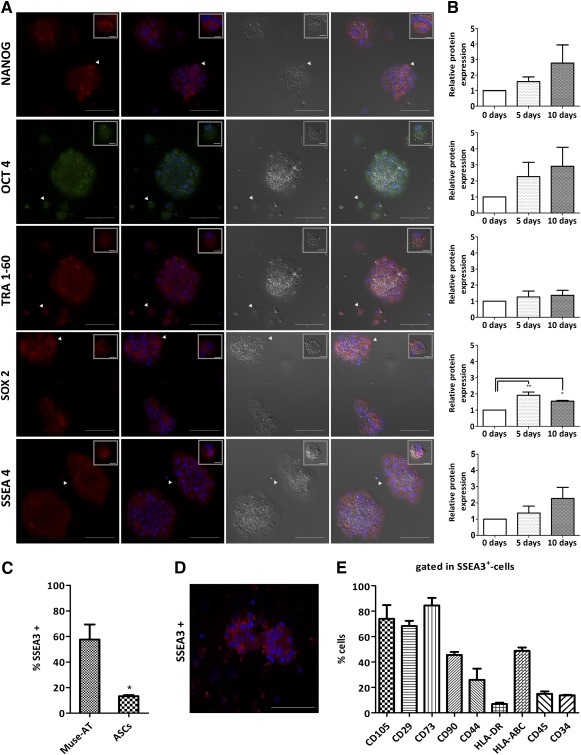
Expression of pluripotency stem cell markers of Muse‐AT cell clusters. **(A):** Representative immunostaining of the stem cell markers Nanong, OCT4, TRA1‐60, Sox‐2, and SSEA‐4 were observed in almost all Muse‐AT cells in the clusters. Scale bar = 100 µm. White arrowheads indicate single cells shown in upper right insets. Scale bar = 5 µm. **(B):** Immunofluorescence quantification of stem cell markers. **(C):** SSEA‐3 was most highly expressed by Muse‐AT cells compared with ASCs, as assessed by fluorescence activated cell sorter (FACS) staining. **(D):** Immunofluorescence staining confirmed SSEA‐3 expression by Muse‐AT cell clusters. Scale bar = 100 µm. **(E):** FACS staining of several CDs revealed the immunophenotype of Muse‐AT cells. *, *p* < .05; **, *p* < .005; *n* = 3 samples analyzed. Abbreviations: ASCs, adipose‐derived stromal cells; d, day; Muse‐AT, multilineage‐differentiating stress‐enduring cells derived from adipose tissue; SSEA, stage‐specific embryonic antigen.

After 10 days in culture, all floating Muse‐AT cells grown in clusters expressed the pluripotency markers assayed as detected by immunofluorescence (Fig. 2A). Muse‐AT cells showed OCT‐4 expression localized in the nuclei, with TRA1‐60 and SSEA‐4 mainly detected at the cell membrane. Additionally, Nanog and Sox‐2 expression was observed in the cytoplasm. Furthermore, pluripotency markers were also detected immediately after isolation (0 days), although at lower levels than after 5 and 10 days in culture (Fig. 2B).

In agreement with previous reports, the SSEA‐3 10 was also expressed in the cell membrane of Muse‐AT cells grown in culture for 7–10 days, as quantified by flow cytometry (57.7% ± 11.8%; *n* = 3) and confirmed by immunofluorescence staining (Fig. 2C, 2D). A small fraction, ∼10% of ASCs also expressed SSEA‐3 on their surface (Fig. 2C). Further immunocytotypic characterization of Muse‐AT cells demonstrated that Muse‐AT‐SSEA‐3^+^ cells expressed CD105 (74% ± 10.8%), CD29 (68.4% ± 4.0%), CD73 (84.5% ± 6.0%), HLA‐ABC (48.7% ± 2.6%), CD44 (26% ± 9.0%), and CD90 (45.5% ± 2.4%) and low levels of CD45, CD34, and HLA‐DR (*n* = 3). Thus, Muse‐AT cells were enriched in the expression of SSEA‐3 at the surface membrane and had a CD expression pattern similar to that of ASCs 26.

### Muse‐AT Cells Differentiate Into the Three Germ Layers

**Figure 3:**
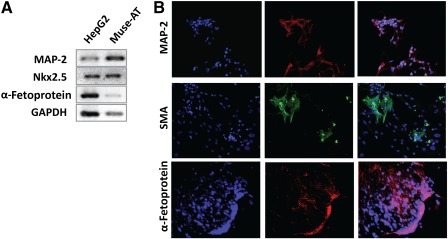
Muse‐AT cells differentiate into the three germ cell lineages. **(A):** Muse‐AT cells cultured in adherent plastic have the ability to spontaneously differentiate into mesodermal, endodermal, and ectodermal lineages, as demonstrated by the expression of mRNAs for Nkx2.5, α‐fetoprotein, and MAP‐2 respectively, determined by qualitative real‐time polymerase chain reaction. **(B):** In the presence of specific differentiation medium, immunofluorescence staining revealed positivity for hepatocyte (α‐fetoprotein, red), myocyte (SMA, green), and neuron (MAP‐2, red) markers. Nuclei were stained with 4′,6‐diamidino‐2‐phenylindole (blue). Original magnification ×200. Abbreviations: GAPDH, glyceraldehyde‐3‐phosphate dehydrogenase; MAP‐2, microtubule‐associated protein 2; Muse‐AT, multilineage‐differentiating stress‐enduring cells derived from adipose tissue; Nkx2.5, NK2 homeobox 5; SMA, smooth muscle actin.

When Muse‐AT cell clusters were seeded onto adherent plastic dishes and cultured in DMEM plus 20% FCS for 7 days without the addition of differentiating factors, they attached to the surface and differentiated. Spontaneous differentiation of Muse‐AT cells into mesodermal, endodermal, and ectodermal lineages was assessed by RT‐PCR amplification of Nkx2.5, α‐fetoprotein, and MAP‐2, respectively (Fig. 3A). Similarly, after culturing Muse‐AT cells under specific defined differentiation media, they expressed specific markers of the three germ layers: α‐fetoprotein (endodermal), SMA (mesodermal), and MAP‐2 (ectodermal), as revealed by immunofluorescence staining (Fig. 3B).

### Muse‐AT Cells Do not Form Teratoma and Show Karyotype Stability

**Figure 4:**
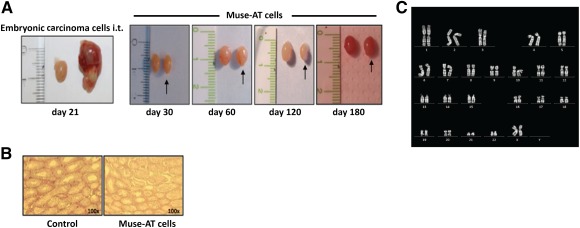
Lack of teratoma formation after transplantation and stability of Muse‐AT cells. **(A):** Muse‐AT cells were injected (10^6^) i.t. into NOD*scid* mice. The transplanted mice were monitored weekly for the appearance of tumors. The P19 embryonic carcinoma cell line was injected (10^6^) as the control. The mice were sacrificed when the tumors became outwardly apparent. NOD*scid* mice injected with Muse‐AT cells did not develop teratoma during the observed period (up to 6 months). **(B):** H&E staining of testes showed normal tissue structure in NOD*scid* Muse‐AT cells of the injected mice. **(C):** Representative normal karyotype of Muse‐AT cells that showed expression of stem cell markers. Abbreviations: d, day; i.t., intratesticular; Muse‐AT, multilineage‐differentiating stress‐enduring cells derived from adipose tissue.

We next injected Muse‐AT cells into immunodeficient mice to evaluate teratoma formation. Transplantation of the P19 mouse embryonic cell line, used as a positive control, into the testes of NOD*scid* mice resulted in teratocarcinoma formation that became outwardly apparent within approximately 20 days. In contrast, Muse‐AT cells (1 × 10^6^)‐injected into the testis of NOD*scid* mice did not develop teratoma during the 6 months of follow‐up (Fig. 4A). Testes that received Muse‐AT cells were recovered at the indicated times, and histological examination after H&E staining revealed normal tissue structure (Fig. 4B). Additionally, independent Muse‐AT cell preparations from three individuals that retained expression of pluripotency markers had normal karyotypes after three culture cycles of floating single cells to suspension clusters (9 days in culture; Fig. 4C).

### Muse‐AT Cells Express High Levels of TGF‐β1

**Figure 5:**
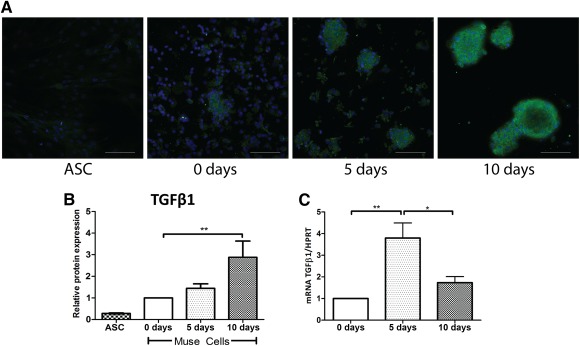
Spontaneous expression of TGF‐β1 by Muse‐AT clusters. **(A):** Immunofluorescence staining of TGF‐β1 at different time points in culture. **(B):** Immunofluorescence quantification showed maximal expression of TGF‐β1 after 10 days in culture. **(C):** Expression levels of TGF‐β1 mRNA of Muse‐AT cell clusters at 0, 5, and 10 days in culture were determined by real‐time polymerase chain reaction. Ct values were normalized to the expression of the *HPRT* gene, and data are expressed relative to the values obtained for day 0. *, *p* < .05; **, *p* < .005; *n* = 5. Abbreviations: ASC, adipose‐derived stromal cell; d, day; Muse‐AT, multilineage‐differentiating stress‐enduring cells derived from adipose tissue; TGF‐β1, transforming growth factor‐β1.

TGF‐β1 is a multifunctional cytokine reported as a promoter or suppressor of tumor cells with immunosuppressive properties. In vitro culture followed by immunofluorescence staining showed that Muse‐AT cells were self‐activated to express high levels of TGF‐β1 (Fig. 5A). Although freshly isolated Muse‐AT cells (0 days) expressed low amounts of immune‐reactive TGF‐β1, longer incubations of Muse‐AT cells in regular culture medium increased TGF‐β1 expression at 5 and 10 days, as quantified by immunofluorescence (Fig. 5A, 5B). Spontaneous expression of TGF‐β1 progressively declined during prolonged periods in culture (data not shown). In line with this observation, TGF‐β1 mRNA levels were increased at 5 days compared with 0 days (fourfold), followed by a significant decline at 10 days in culture (Fig. 5C). In contrast, ASCs expressed very low immune‐reactive TGF‐β1 at 5–10 days in culture (Fig. 5A, 5B).

### Muse‐AT Cells Have Immunomodulatory Properties

**Figure 6:**
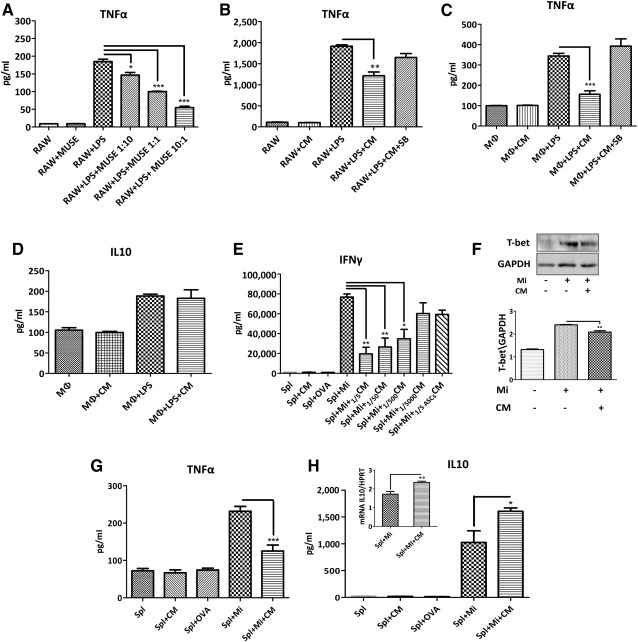
Muse‐AT cell activity on LPS‐stimulated macrophages and splenocytes. **(A):** Muse‐AT cells in coculture with the RAW macrophage cell line. RAW cells were seeded onto the lower chamber of a Transwell plate (Corning) and stimulated with LPS for 20 minutes. LPS was removed, the cells washed, and Muse‐AT cells were then cultured (RAW/Muse‐AT in a 1:10; 1:1, and 10:1 ratio) in the upper chamber. **(B):** Muse‐AT cell CM effects on LPS‐stimulated RAW cells with and without the presence of SB 431542 (SB; 10 ng/ml). **(C, D):** Freshly isolated murine peritoneal MΦ stimulated with LPS. The effect of Muse‐AT cell CM on the secretion of TNF‐α **(C)** and IL‐10 **(D)** was evaluated with and without SB in the culture medium. **(E):** Antigen‐specific T‐cell response of NOD BDC2.5 splenocytes in culture. Muse‐AT cell CM was assayed at different dilutions (1 to 5 to 1 to 5,000; *n* = 3). **(F):** Muse‐AT cell CM downregulated antigen‐stimulated T‐bet expression assessed by Western blot (*n* = 3). **(G):** Inhibitory effect on antigen‐challenged T cells on TNF‐α secretion by Muse‐AT cell CM (*n* = 3). **(H):** Muse‐AT cell CM enhanced anti‐inflammatory cytokine IL‐10 secretion in antigen‐stimulated T cells (*n* = 6). The expression levels of mRNA IL‐10 relative to mRNA HPRT are indicated as an inset. Cytokine levels were quantified in 72‐hour culture supernatants by enzyme‐linked immunosorbent assays. OVA was used as a control. Mi concentration = 5 nM. *, *p* < .05; **, *p* < .005; ***, *p* < .0005. Abbreviations: _ASCs_CM, adipose stem cell conditioned media; CM, conditioned media; GAPDH, glyceraldehyde‐3‐phosphate dehydrogenase; IL, interleukin; LPS, lipopolysaccharide; MΦ, macrophages; Mi, mimotope; Muse‐AT, multilineage‐differentiating stress‐enduring cells derived from adipose tissue; NOD, nonobese diabetic; OVA, ovalbumin; Spl, splenocytes; T‐bet, T‐box transcription factor (TBX21); TNF‐α, tumor necrosis factor‐α.

It has been reported that MSCs have immunomodulatory properties 27. To investigate whether Muse‐AT cells might influence an immune response, we first used the mouse macrophage RAW cell line to study putative immunomodulatory activity. It is well known that LPS activates and dramatically induces the secretion of TNF‐α by RAW cells 28. Muse‐AT cells cocultured with RAW macrophages significantly reduced TNF‐α secretion on LPS stimulation (data not shown). Thus, we tested the hypothesis that soluble signals released from Muse‐AT cells would be the mediators of the observed reduced LPS‐stimulated TNF‐α secretion. RAW cells were plated on the bottom well of a 24‐well Transwell (3‐µm pore size) coculture system (Corning, Corning, NY, http://www.corning.com), stimulated with LPS or not (control), and cultured with Muse‐AT cells seeded at several cell densities (Fig. 6A) onto the upper well during 24 hours. The presence of Muse‐AT cells in coculture largely decreased the secretion of TNF‐α by RAW cells (up to approximately fourfold; *p* < .0005, RAW/Muse 10:1 vs. control; Fig. 6A). This result indicates that the soluble mediators secreted by Muse‐AT cells might be, in part, responsible for this effect. Similarly, Muse‐AT cell CM at 1 to 5 dilution significantly reduced TNF‐α secretion by LPS‐stimulated RAW cells (*p* < .005 vs. control; Fig. 6B). To confirm whether the action of Muse‐AT cell CM on RAW cells might be due to its TGF‐β1 content, we sought to block its signaling pathway using a small molecule inhibitor of the type I TGF‐β receptor, SB‐431542 (SB). When SB was applied to LPS‐stimulated RAW cultures, the CM did not perturb the production of TNF‐α (Fig. 6B). IL‐10 secretion by RAW cells was not detected (data not shown). To confirm these results with a more reliable source of macrophages, we used freshly isolated murine macrophages (MΦ). We obtained similar results, with Muse‐AT cell CM reducing TNF‐α secretion by LPS‐stimulated MΦ (*p* < .05 vs. control; Fig. 6C), and the incorporation of SB into the media restored TNF‐α secretion. The secretion of IL‐10 by LPS‐stimulated MΦ was not changed by the influence of the Muse‐AT cell CM (Fig. 6D).

We then examined whether Muse‐AT cells would be able to influence the T lymphocyte response. We used an antigen‐specific T‐lymphocyte in vitro assay based on BDC2.5 CD4^+^ T cells that specifically recognized a chromogranin‐A peptide in the context of NOD major histocompatibility complex class II complex (H‐2^g7^). BDC2.5 CD4^+^ T lymphocytes present a Th1 phenotype secreting high amounts of IFN‐γ and TNF‐α on antigen stimulation and have been extensively characterized with respect to pathogenicity in autoimmune diabetes 21. Thus, BDC2.5 CD4^+^ T cells responded with a high secretion of INF‐γ when stimulated with a mimotope of chromogranin A peptide (5 nM) for 72 hours 22, 29. As expected, BDC2.5 CD4^+^ T cells did not respond to ovalbumin (OVA) (negative control), demonstrating the specificity of our bioassay (Fig. 6E). Muse‐AT cell CM diminished IFN‐γ secretion under the same culture conditions in a concentration‐response manner. The addition of CM diluted 1 to 5 (_1/5_CM) to the culture of mimotope‐stimulated BDC2.5 splenocytes resulted in significant reduction of IFN‐γ secretion (fourfold vs. mimotope‐stimulated splenocytes; *p* < .005). Both ASC‐_1/5_CM and Muse‐AT cell‐_1/5,000_CM showed slightly diminishing activity on IFN‐γ secretion by BDC2.5 splenocytes, although the difference was not statistically significant compared with mimotope‐stimulated splenocytes (Fig. 6E). Therefore, we chose Muse‐AT cell‐_1/5_CM for further studies. Control experiments replacing Muse‐AT cell CM with fresh medium with or without serum showed that the effect on IFN‐γ secretion was not due to exhaustion of the culture medium (data not shown). TBX21 (T‐box transcription factor, also known as T‐bet) governs Th1 differentiation and the expression of IFN‐γ 30. Therefore, we questioned whether Muse‐AT cells might be able to influence T‐bet expression. Western blot analysis indicated that Muse‐AT cell CM reduced the expression of T‐bet by antigen‐specific stimulated BDC2.5 splenocytes (Fig. 6F), indicating that low levels of IFN‐γ secretion might be due in part to reduced expression of T‐bet.

Subsequently, we investigated the antigen‐specific secretion of proinflammatory TNF‐α secreted by BDC2.5 NOD splenocytes. The TNF‐α levels in culture supernatant were significantly lower (*p* < .005) in the presence of _1/5_CM compared with mimotope‐stimulated splenocytes (Fig. 6G). On antigen stimulation, naïve CD4^+^ T lymphocytes can differentiate into different Th effector subpopulations. BDC2.5 NOD CD4^+^ T cells have a marked Th1 bias, as previously reported 21. We measured the effect of Muse‐AT cell CM on the secretion of IL‐10, a well‐known anti‐inflammatory cytokine. Muse‐AT cell CM augmented IL‐10 secretion in mimotope‐stimulated BDC2.5 NOD splenocytes (*p* < .05; Fig. 6H). Moreover, mRNA IL‐10 expression levels increased in mimotope‐stimulated BDC2.5 NOD splenocytes (*p* < .005; Fig. 6H, inset). Intracellular cytokine (IFN‐γ, IL‐4, IL‐5, IL‐10) levels in CD4^+^ T lymphocytes did not show changes for any condition after splenocyte challenge (data not shown). Also, Muse‐AT cell CM did not affect CD4^+^ T‐lymphocyte proliferation ( supplemental online Fig. 1). However, the expression of GATA‐3 mRNA levels, a master transcription factor for Th2 differentiation, was not influenced by Muse‐AT cell CM (data not shown). Altogether, the data obtained suggest that the diminution of the Th1 profile might result mainly from reduced activity of transcription factors such as T‐bet, responsible for Th1 type commitment and not from inhibition of Th1 proliferation.

### Effects of Muse‐AT Cells in Cytokine Secretion Are Mediated Through TGF‐β1/pSMAD2 Signaling Pathway

**Figure 7:**
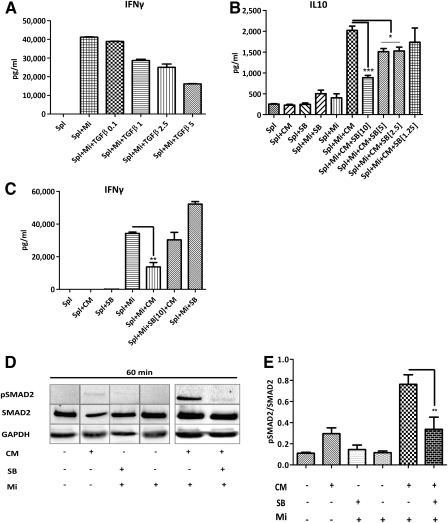
TGF‐β1 signaling blockade on cytokine secretion. **(A):** Exogenously added TGF‐β1 to antigen‐stimulated splenocytes showed a concentration response dependence on IFN‐γ secretion. **(B):** The effect of TGF‐β1 signaling blockade was assessed using SB at different concentrations (1.25–10 ng/ml) on antigen‐challenged T cells followed IL‐10 quantification. **(C):** Inhibition of TGF‐β1 signaling with 10 ng/ml of SB restored IFN‐γ secretion levels. **(D):** SB diminished pSMAD2 levels assessed by Western blot (WB) in antigen‐stimulated T cells. **(E):** Quantification of WB analysis shown in **(D)**. Cytokine measured by enzyme‐linked immunosorbent assays in 72‐hour supernatants. *, *p* < .05; **, *p* < .005; ***, *p* < .0005; *n* = 3. Abbreviations: CM, conditioned media; GAPDH, glyceraldehyde‐3‐phosphate dehydrogenase; IFN‐γ, interferon‐γ; Mi, mimotope; Muse‐AT, multilineage‐differentiating stress‐enduring cells derived from adipose tissue; p, phosphorylated; SB, SB 431542; Spl, splenocytes; TGF‐β1, transforming growth factor‐β1.

To further investigate whether the molecular mechanisms of Muse‐AT cells on cytokine secretion involve the TGF‐β1 signaling pathway, we first assayed its action on the secretion of IFN‐γ by antigen‐specific stimulated BDC2.5 splenocytes. IFN‐γ was significantly reduced by exogenous TGF‐β1 in a concentration‐dependent manner, reaching the lowest level of secretion at 5 ng/ml (Fig. 7A). Therefore, to confirm whether the action of Muse‐AT cell CM on T cells might result from increased TGF‐β1 secretion levels, we sought to block its signaling pathway using SB. When SB was applied to splenocyte cultures, the Muse‐AT cell CM reduced the production of IL‐10 (approximately twofold at 10 mM SB and 1.3‐fold at 2.5–5 mM SB; Fig. 7B) and restored the secretion of IFN‐γ on antigen‐specific stimulated BDC2.5 splenocytes (Fig. 7C). The addition of SB increased the secretion of mimotope‐stimulated IFN‐γ, suggesting that a putative blockade of splenocyte‐derived TGF‐β1 might also contribute to IFN‐γ secretion. Thus, in the presence of CM, SB inhibited the activity of TGF‐β1 from both sources (i.e., Muse‐AT CM and splenocytes). TGF‐β ligands bind to specific receptors to initiate the intracellular signaling cascade from the cytoplasm to the nucleus by phosphorylating mediators, mainly a family of proteins called SMADs, specifically SMAD2/3. SMAD2 has been described as an important mediator of TGF‐β1 anti‐inflammatory activity 31. Thus, we tested pSMAD2 levels as an intracellular mediator of TGF‐β1 action on antigen‐specific stimulation of BDC2.5 splenocytes. As expected, the addition of exogenous TGF‐β1 increased the amount of pSMAD2 produced by BDC2.5 splenocytes, reaching its maximum level at 30–60 minutes after stimulation (data not shown). Similarly, CM from Muse‐AT cells also increased SMAD2 phosphorylation on antigen‐specific stimulated BDC2.5 splenocytes that was abolished by SB treatment (Fig. 7D, 7E). Collectively, these data suggest that the immunomodulatory activity exerted by Muse‐AT cell CM on cytokine secretion by antigen‐challenged BDC2.5 splenocytes might be attributable to the TGF‐β1/pSMAD2 signaling pathway.

## Discussion

Several reports have described the therapeutic potential of stem cells obtained from various origins. Among them, MSCs are the most popular because of the simple isolation methods and their capacity to induce in vivo the expression of cytokines and other biologics with a wide spectrum of action 32, 33. Although MSCs have been evaluated in many clinical studies, their use as a trustworthy medical practice is still awaited. One of the major limitations in the use of MSCs in regenerative medicine is the low survival rate (1%–3%) after transplantation 34, 35. Muse cells, a novel pluripotent stem cell population present in low amounts in MSCs 9 were originally identified as stress‐tolerant cells and were isolated using methods that took advantage of their SSEA‐3 expression 9. In the present study, we have documented the isolation of Muse‐AT cells under severe cellular stress conditions as a highly enriched SSEA‐3‐expressing cell population (∼60%), which grew as aggregates at 7–10 days in culture. Compared with pluripotent cells from other origins, Muse‐AT cells might represent a more realistic source of stem cells for regenerative medicine. Their potential medical use is because they can be obtained at low cost after a simple procedure from accessible and abundant adult tissue, such as lipoaspirates. Unlike other well‐established pluripotent cells, Muse‐AT cells are able to differentiate into all germ layers without teratoma formation in vivo. Additionally, Muse‐AT cells can be obtained directly from adult tissues avoiding the ethical concerns arising with ES cell procurement and genetically modified iPSCs.

It has been demonstrated that culture of MSCs as three‐dimensional spheroids/aggregates can be obtained using different protocols and that this method increases their therapeutic potential 36 37 38. MSC growth into three‐dimensional spheroids enhances their immune‐modulating properties by expressing high levels of anti‐inflammatory proteins 36. MSCs grown as spheroids are smaller than in those grown in adherent conditions. The Muse‐AT cells had a small diameter and exhibited a high nuclear/cytoplasm ratio typical of ES cells, as we observed by electron microscopy. In contrast, it has been described that MSCs do not easily form aggregates unless cultured in dynamic three‐dimensional conditions 37. In the present study, we showed that Muse‐AT cells spontaneously aggregate when cultured in nonadherent plastic cell culture vessels, without the addition of specific growth factors or extracellular matrices, and undergo limited proliferation after cycles of disaggregation/aggregation. Freshly isolated Muse‐AT cells expressed pluripotency stem cell markers at all times under our culture conditions without the addition of specific factors. We believe this phenomenon can be attributed to the formation of cell clusters that facilitate cell to cell contacts with natural extracellular matrix components. This might help to maintain the “stemness” of Muse‐AT cells and to improve their differentiation, just as it has been shown for three‐dimensional culture of MSCs 39. Muse‐AT cells in clusters maintained the surface expression of several CDs often used as MSC markers that facilitate cell‐cell and cell‐extracellular matrix contacts 40. We have also demonstrated that Muse‐AT cells do not form teratomas, just as was described for Muse cells obtained from bone marrow and dermal fibroblasts 41. The pleiotropic cytokine TGF‐β1 has been proposed as a stemness regulator of hematopoietic stem cells (HSCs) through phosphorylation of SMAD2, an intracellular transducer acting downstream of the TGF‐β receptors 42. HSCs require stimulation with activin‐like kinase 5 (TGF‐β type I receptor) ligands to maintain a quiescent state in the bone marrow niche. In accordance, we observed that the increment of TGF‐β1 levels in spheroid Muse‐AT cells also matched, with very low proliferation cell activity. In contrast, we observed very low TGF‐β1 levels in highly proliferative ASCs. Thus, our observations led us to speculate that the TGF‐β autocrine/paracrine loop might help to maintain Muse‐AT cells at a low proliferation stage 9. Freshly isolated Muse‐AT cells express the TGF‐β receptor (M.L. Gimeno, F. Fuertes, L. Ariolfo et al., unpublished observation), suggesting that they might respond early to TGF‐β ligands. In this regard, Ylöstalo et al. proposed that aggregation of MSCs into spheroids induces a type of cellular stress that results in intracellular signals that lead to upregulation of anti‐inflammatory effectors 43. However, activation might occur in vivo by action of soluble factors released from injured tissues 44. Accordingly, we found that Muse‐AT cells spontaneously expressed high levels of TGF‐β1 when cultured in nonadherent conditions (e.g., 5–10 days) and that cell aggregation might trigger TGF‐β1 expression.

In addition to being attractive for tissue repair, interest has increased in the use of MSCs because of both their immunomodulatory capacity and their ability to home to sites of inflammation and injury. The immunomodulatory functions of MSCs have been described in terms of their interaction with various immune cells such as T and B lymphocytes, dendritic cells, and natural killer cells by the expression and secretion of several soluble factors and/or through direct cell‐to‐cell contact‐dependent mechanisms 45. These characteristics have attracted the interest of researchers to use MSCs in animal models of several immune‐related diseases such as rheumatoid arthritis, systemic lupus erythematosus, inflammatory bowel disease, and atopic dermatitis and transplantation 46 47 48 49 50. The question regarding whether Muse‐AT cells possess immunoregulatory capacities has remained largely untouched until now. We have provided evidence for the first time that Muse‐AT cells significantly diminish the secretion of the proinflammatory cytokine TNF‐α by LPS‐stimulated RAW cells and peritoneal murine macrophages. Additionally, we found that they modulated the balance of cytokines secreted by antigen‐challenged T cells toward a reduction of the secretion of proinflammatory cytokines (IFN‐γ and TNF‐α) with increasing IL‐10 (anti‐inflammatory) production. In the same line of evidence, it has previously been reported that MSCs grown as spheroids showed anti‐inflammatory capacity and converted macrophages to an anti‐inflammatory phenotype through the expression of tumor necrosis factor‐inducible gene 6 (TSG‐6) 36 or prostaglandin E_2_ 43, 44. We found that as soon as Muse‐AT cells had aggregated in culture, they began to increase TGF‐β1 expression. Mechanistically, we documented that the TGF‐β1 produced by Muse‐AT cells might be pivotal for the modulation of cytokine secretion by macrophages and antigen‐mediated T‐cell responses. It is known that MSCs also produce many paracrine mediators and cytokines, such as TGF‐β1 51, 52. In agreement with that, we found that ASCs also produced TGF‐β1, although at low levels and with a minor anti‐inflammatory effect on antigen‐stimulated T lymphocytes. It has been reported that MSCs might protect breast cancer cells through the secretion of TGF‐β that induces increases in regulatory T cells 53. Although we do not exclude the possibility that Muse‐AT cells might generate T lymphocytes with a regulatory phenotype, we found that they did decrease the expression of T‐bet on antigen‐challenged T lymphocytes, demonstrating that this transcriptional factor is involved in the observed modulation of IFN‐γ secretion. Also, we could not exclude the possibility that Muse‐AT cell CM might indirectly regulate the T‐cell response by modulating myeloid lineage cells.

Moreover, Muse‐AT cell CM induced the expression of pSMAD2, an effect blocked by a type I TGF‐β receptor inhibitor, suggesting that TGF‐β1 is a prominent actor of this effect. The key role of TGF‐β1 in immunoregulation is well known. TGF‐β1 knockout mice die of overwhelming inflammation 54. Strong evidence has shown that TGF‐β1 inhibition upregulates T‐bet and increases Th1 cytokines in the human gut 55. Similarly, our results showed upregulation of pSMAD2, decreased expression of T‐bet, and the consequent impairment of IFN‐γ secretion by Muse‐AT cell CM. We have documented in the present study that TGF‐β1 appears to be critical in Muse‐AT cell‐associated immunomodulation. Abrogation of the TGF‐β anti‐inflammatory effect by an inhibitor of type I TGF‐β receptor led us to confirm the role of TGF‐β1 in this rather unique immunoregulatory capacity of Muse‐AT cells.

## Conclusion

Our data have shown, for the first time, the immunomodulatory capacity of Muse‐AT cells is mainly based on TGF‐β action over immune cells in a pSMAD2‐dependent manner. As demonstrated, pluripotent Muse‐AT cells can be easily obtained from human lipoaspirates, taking advantage of their stress‐enduring features and avoiding cell sorting or genetic manipulation methods. Muse‐AT cells could represent a novel immunomodulatory tool for the treatment of inflammatory immune‐related disorders.

## Author Contributions

M.L.G.: conception and design, collection and/or assembly of data, data analysis and interpretation; F.F., A.E.B.T., A.I.A., and T.C.O.: collection and/or assembly of data; R.C. and L.C.: provision of study material or patients; M.C.S., L.L., and R.A.D.: data analysis and interpretation; M.J.P.: conception and design, collection and/or assembly of data, data analysis and interpretation, financial support, administrative support, manuscript writing, final approval of manuscript.

## Disclosure of Potential Conflicts of Interest

The authors indicated no potential conflicts of interest.

## Supporting information

Supporting Information

Supporting Information
