# Supplementary figures and images for: Pluripotent Nontumorigenic Adipose Tissue‐Derived Muse Cells have Immunomodulatory Capacity Mediated by Transforming Growth Factor‐β1
Source: Stem Cells Transl Med. 2016 Aug 2;6(1):161–73. doi: 10.5966/sctm.2016-0014 (PMC5442729; doi:10.5966/sctm.2016-0014)

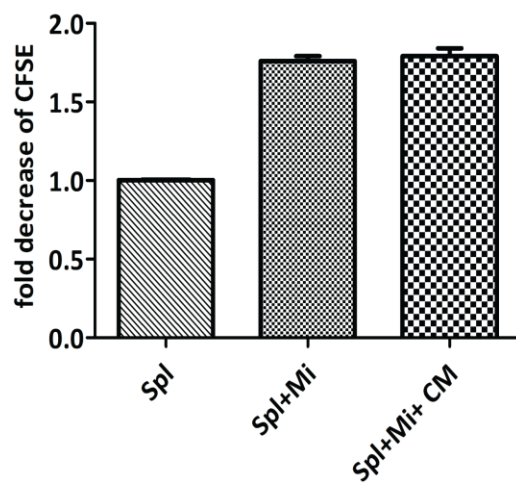

Muse-AT cells -CM did not disturb CD4+ T cells proliferation determined by CFSE dilution, n=3

Supplement: Supplementary file 1 — Supporting Information [file SCT3-6-161-s001.pdf]
